# Effect of Al Content on the Wear Evolution of Ti_1-x_Al_x_N-Coated Tools Milling Ti-6Al-4V Alloy

**DOI:** 10.3390/mi14061228

**Published:** 2023-06-10

**Authors:** Guanghui Fan, Jingjie Zhang, Peirong Zhang, Jin Du, Chonghai Xu, Mingdong Yi, Guoqing Zhang

**Affiliations:** 1 Faculty of Mechanical Engineering, Qilu University of Technology (Shandong Academy of Sciences), Jinan 250353, China; 10431200002@stu.qlu.edu.cn (G.F.); zhangpeirong2012@163.com (P.Z.); dj84105@qlu.edu.cn (J.D.); xch@qlu.edu.cn (C.X.); yimingdong@qlu.edu.cn (M.Y.); 10431200063@stu.qlu.edu.cn (G.Z.); 2 Key Laboratory of Equipments Manufacturing and Intelligent Measurement and Control, China National Light Industry, Qilu University of Technology (Shandong Academy of Sciences), Jinan 250353, China

**Keywords:** Ti_1-x_Al_x_N-coated tools, Ti-6Al-4V alloy, evolution of wear form, wear mechanism, milling

## Abstract

Ti_1-x_Al_x_N coating is formed by replacing some Ti atoms in TiN with Al atoms, and their properties are closely related to Al content (0 < x < 1). Recently, Ti_1-x_Al_x_N-coated tools have been widely used in the machining of Ti-6Al-4V alloy. In this paper, the hard-to-machine material Ti-6Al-4V alloy is used as the study material. Ti_1-x_Al_x_N-coated tools are used for milling experiments. The evolution of the wear form and the wear mechanism of Ti_1-x_Al_x_N-coated tools are studied, and the influence of Al content (x = 0.52, 0.62) and cutting speed on tool wear are analyzed. The results show that the wear on the rake face changes from the initial adhesion and micro-chipping to coating delamination and chipping. Wear on the flank face varies from the initial adhesion and grooves to boundary wear, build-up layer, and ablation. The main wear mechanisms of Ti_1-x_Al_x_N-coated tools are dominated by adhesion, diffusion, and oxidation wear. Ti_0.48_Al_0.52_N coating protects the tool well and extends its service life.

## 1. Introduction

Ti-6Al-4V alloy is a common α + β-type titanium alloy with good overall performance which is often used in the biomedical [1,2] and aerospace fields [3,4]. However, its poor thermal conductivity, low elastic modulus, and high chemical activity make Ti-6Al-4V alloy typical of hard-to-machine materials. During the machining of Ti-6Al-4V alloy, excessive cutting force, high cutting temperature, and severe tool wear often occur [5,6].

The tool coating acts as a thermal and chemical barrier, slowing down the oxidation and element diffusion of the tool material. Tool coating also improves tool resistance to wear and adhesion, allowing for longer tool service life and higher-speed machining applications [7]. Having excellent performance, coated tools are widely used to cut various materials [8,9,10], accounting for 80% of the total tool count. Among them, Ti_1-x_Al_x_N-coated tools are widely used in titanium alloy machining [11,12,13] due to their high heat resistance, superior chemical resistance, and low friction coefficient [14,15]. Therefore, extensive studies have been conducted on the performance and wear of Ti_1-x_Al_x_N-coated tools.

The researchers found that the structure of Ti_1-x_Al_x_N coating was associated with the Al content x. When 0 < x ≤ 0.52, the coating is composed of Ti_1-x_Al_x_N (002) grains with a single phase B1-NaCl structure. When 0.52 < x ≤ 0.59, the coating is composed of Ti_1-x_Al_x_N (002) grains with a B1-NaCl structure and AlN-rich Ti_1-x_Al_x_N (0002) and (10$\overset{-}{1}$1) grains with a wurtzite structure. When 0.59 < x ≤ 0.86, the coating is composed of AlN-rich Ti_1-x_Al_x_N (0002) and (10$\overset{-}{1}$1) grains with a wurtzite structure. When x = 1, the coating is composed of AlN (0002) grains with a single phase wurtzite structure [16]. The Al content affects the coating structure, and changes in the coating structure induce changes in the mechanical properties of the Ti_1-x_Al_x_N coating. The effect of Al content on the performance of Ti_1-x_Al_x_N coating has been extensively studied by researchers [17]. The hardness and elastic modulus of the Ti_1-x_Al_x_N coating showed an increasing trend, with increasing Al content followed by a decreasing trend [18]. When x = 0.52, 0.62, the Ti_1-x_Al_x_N coating has high hardness. The wear resistance is better when the coating hardness is higher. The toughness of the Ti_1-x_Al_x_N coating showed a trend of increasing and then decreasing with increasing Al content. Among them, Ti_0.47_Al_0.53_N coating had the best toughness [19]. In addition, Al content had an important effect on the thermal conductivity [20] and oxidation resistance [21] of the Ti_1-x_Al_x_N coating. The Ti_1-x_Al_x_N coating showed a trend of increasing and then decreasing thermal conductivity with increasing Al content. Additionally, a minimum thermal conductivity of about 4.63 W/mK was obtained at the Al content x = 0.42.

In titanium alloy machining, the Ti_1-x_Al_x_N-coated tools exhibited better machining quality and better service life than uncoated carbide tools [22,23,24]. Chang’s [25] research showed that crater, ablation, and scratches were the main wear forms on Ti_1-x_Al_x_N-coated tools during the turning of Ti-6Al-4V at a cutting speed of 110 m/min, feed rate of 0.2 mm/rev, and depth of 0.3 mm. Adhesion wear and oxidation wear were the main wear mechanisms. Surface hardness and residual stress have an important effect on the cutting performance of Ti_1-x_Al_x_N-coated tools. Ti_1-x_Al_x_N-coated tools had better wear resistance and longer service life, with low roughness, high surface hardness, and medium residual stress. High residual stresses force cracks to be expanded along the plane, which could lead to coating delamination and the formation of a smooth, worn surface. Smooth wear surfaces can reduce contact pressure, slowing tool wear and extending tool life [26]. Additionally, lower surface roughness can reduce the generation of cutting heat during machining and slow down tool wear. The researchers also reduce the surface roughness by preprocessing the cutting edge, thereby improving the cutting performance [27]. The results of the Msic A [28] study indicated that the main wear forms of Ti_1-x_Al_x_N-coated tools were BUE and crater when the titanium alloys were turned at a cutting speed of 150 m/min, feed rate of 0.1225 mm/rev, and depth of cut of 0.25 mm, with adhesion wear and diffusion wear being the main wear mechanisms. Wang [29] found that flank face wear, chipping, and coating delamination during the titanium milling were the main forms of tool failure in Ti_1-x_Al_x_N-coated tools. The same analysis was used in the study by Hou [26]. To further reveal the tool wear, the researchers analyzed the wear evolution of Ti_1-x_Al_x_N-coated tools. Chang’s [30] study showed that Ti_1-x_Al_x_N-coated tools underwent BUE formation, coating delamination, and crater wear before reaching failure. Additionally, the final tool failure was due to chipping. Wang [11] analyzed the wear evolution of Ti_1-x_Al_x_N-coated tools using its microstructural features. The coated tool underwent the process of coating cracking and delamination before reaching the end of its service life.

Most of the current research on Ti_1-x_Al_x_N-coated tools wear in titanium alloy cutting has focused on tool failure. To clarify the wear process of Ti_1-x_Al_x_N-coated tools, it is important to investigate the evolution of the wear form and the wear mechanism of Ti_1-x_Al_x_N-coated tools before the failure of the tool. Additionally, it is worthwhile to investigate the effect of Al content and cutting speed on the wear evolution of Ti_1-x_Al_x_N-coated tools.

In this paper, Ti_1-x_Al_x_N-coated tools with different Al contents were used to mill Ti-6Al-4V alloy to study the wear forms and wear mechanisms at different stages. The wear evolution of the Ti_1-x_Al_x_N-coated tools was also analyzed. Meanwhile, the influence of the Al content and cutting speed on the wear evolution of Ti_1-x_Al_x_N-coated tools were analyzed. This study can provide a theoretical basis for the study of tool wear in Ti_1-x_Al_x_N-coated tools for titanium alloy milling. It can better guide cutting, slow down tool wear, and extend tool service life.

## 2. Experimental Details

Ti_1-x_Al_x_N (x = 0.52, 0.62)-coated tools were obtained using PVD technologies and were produced by Zhuzhou DIA. Ti-6Al-4V alloy was selected as the workpiece material with the dimensions of 70 × 70 × 100 mm. The characterization tests of the Ti_1-x_Al_x_N-coated tools and Ti-6Al-4V alloy are shown as follows.

### 2.1. Surface Analysis of Ti_1-x_Al_x_N-Coated Tools

**Figure 1:**
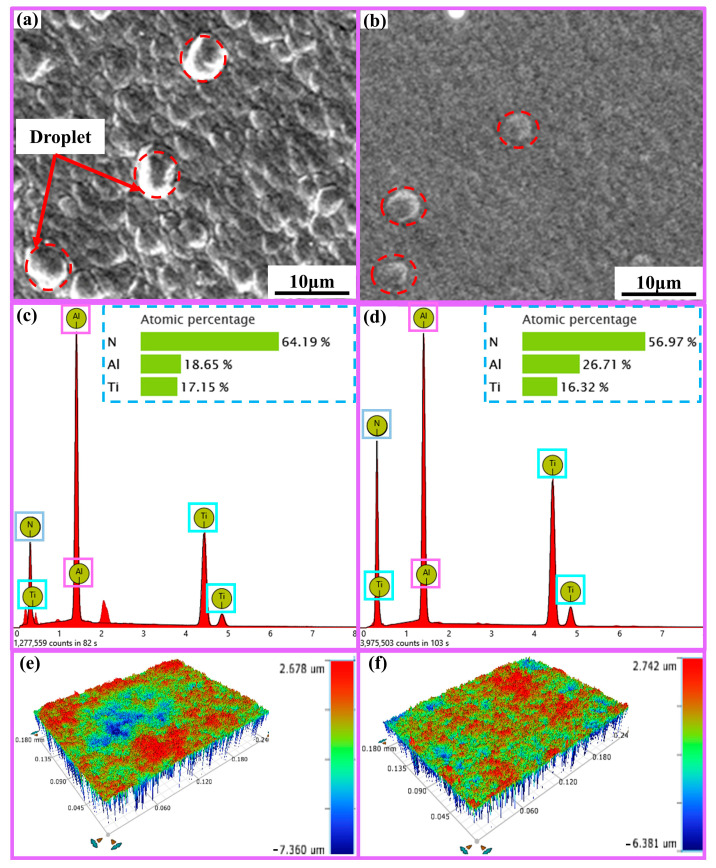
Surface analysis of Ti_1-x_Al_x_N-coated tools: (**a**) Ti_0.48_Al_0.52_N surface morphology; (**b**) Ti_0.38_Al_0.62_N surface morphology; (**c**) EDS analysis of Ti_0.48_Al_0.52_N-coated tool surface; (**d**) EDS analysis of Ti_0.38_Al_0.62_N-coated tool surface; (**e**) Ti_0.48_Al_0.52_N-coated tool surface 3D morphology; (**f**) Ti_0.38_Al_0.62_N-coated tool surface 3D morphology.

SEM (Phenom Prox, Funa Scientific Instrument Co., Ltd., Shanghai, China) and 3D optical microscope (Contour Elite K, Brook Scientific Instruments, Madison, WI, USA) analyses were used to characterize the Ti_1-x_Al_x_N tools’ surface morphology, surface three-dimensional morphology, and elemental composition, as shown in Figure 1. The surface roughness values were obtained using the 3D morphology analysis of the Ti_1-x_Al_x_N-coated tools. The surface roughness of the Ti_0.48_Al_0.52_N-coated tool (0.327 ± 0.012 μm) was higher than the Ti_0.38_Al_0.62_N-coated tool (0.285 ± 0.011 μm). As a result, the friction coefficient between the Ti_0.48_Al_0.52_N-coated tool and the workpiece material was higher. This resulted in more cutting force and a higher cutting temperature during the cutting process.

### 2.2. Ti_1-x_Al_x_N-Coated Tools Nanoindentation and Scratch Test

**Figure 2:**
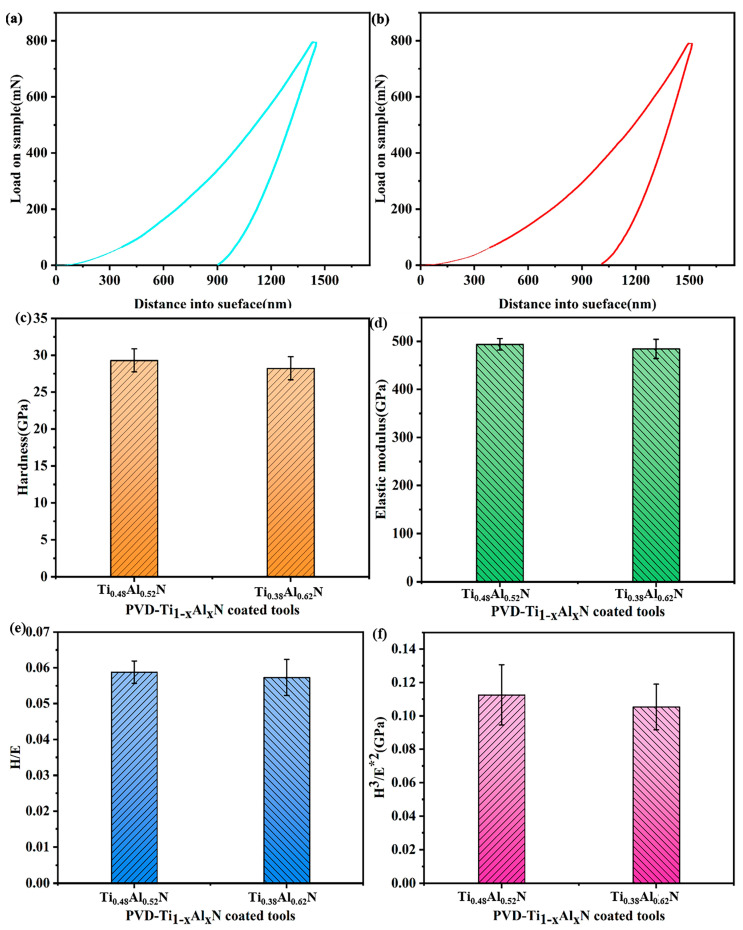
Indentation test of Ti_1-x_Al_x_N-coated tools: (**a**) Ti_0.48_Al_0.52_N-coated tool load–displacement curve; (**b**) Ti_0.38_Al_0.62_N-coated tool load–displacement curve; (**c**) coating hardness; (**d**) coating elastic modulus; (**e**) H/E; (**f**) H^3^/E*^2^.

The hardness and elastic modulus of the Ti_1-x_Al_x_N coating were measured using a G200 Nano Indenter (Keysight, Santa Rosa, CA, USA), and the load–displacement curves of the Ti_1-x_Al_x_N coatings were obtained as shown in Figure 2a,b. The hardness and elastic modulus of the coating should be measured in such a way that the indentation depth of the hardness tester does not exceed 1/10 of the coating thickness (<300 nm). Therefore, an interval of 100–200 nm of the load–displacement curve was selected to calculate the hardness and elastic modulus of the coatings, as shown in Figure 2c,d. H/E and H^3^/E*^2^ (E*^2^ = E×(1–ν^2^), ν is the Poisson’s ratio of the coating, which is taken as 0.22) were calculated from the hardness and elastic modulus of the coating, as shown in Figure 2e,f.

As shown in Figure 2c,d, the hardness of the Ti_0.48_Al_0.52_N coating (29.3 GPa) was higher than that of the Ti_0.38_Al_0.62_N coating (28 GPa). The elastic modulus of the Ti_0.48_Al_0.52_N coating (494 GPa) was higher than that of the Ti_0.38_Al_0.62_N coating (484 GPa). Additionally, the increase in Al content decreased the hardness and elastic modulus of the coating. When x = 0.62, the formation of the soften AlN phase with lower hardness in the coating led to the hardness of the coating [31]. Additionally, the formation of the AlN phase had an effect on elastic modulus [18]. As shown in Figure 2e,f, the Ti_0.48_Al_0.52_N coating had higher H/E and H^3^/E*^2^. High H/E and H^3^/E*^2^ can increase the wear resistance of the coating [32].

**Figure 3:**
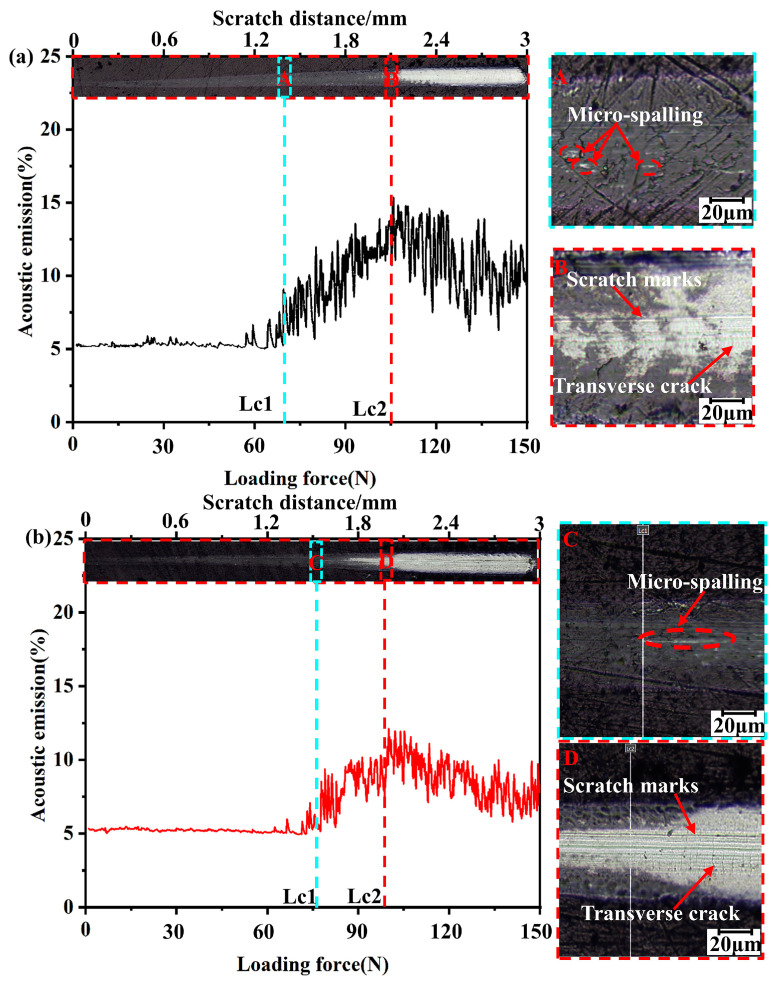
Scratch test results of Ti_1-x_Al_x_N-coated tools: (**a**) Ti_0.48_Al_0.52_N-coated tool; (**b**) Ti_0.38_Al_0.62_N-coated tool.

The scratch test of the Ti_1-x_Al_x_N-coated tools was performed using a high-load scratch instrument (Revetest RST3, Basel, Switzerland). The scratch morphology of the Ti_1-x_Al_x_N-coated tools are shown in Figure 3. *Lc1*, *Lc2*, and *CPRs* are shown in Table 1.

The scratch crack propagation resistance (*CPRs*) was calculated using Equation (1) and can be qualitatively expressed as toughness [33]:

(1) CPRs=Lc1 (Lc2−Lc1)

where *Lc*1 indicates the initial peeling point load and *Lc*2 indicates the critical load at which the delamination and adhesion failure of the coating occurs.

Ti_1-x_Al_x_N coating is a plastic coating, so its critical load is determined by *Lc1* and *Lc2*. As shown in Figure 3a,b, the acoustic signal at *Lc*1 showed a sharp increase and the coating exhibited a micro-spalling phenomenon. As the load gradually increased, the acoustic signal at *Lc*2 showed a sudden decrease. The coating showed a large area of delamination and the tool substrate was exposed to *Lc*2. At the same time, Ti_1-x_Al_x_N-coated tools showed obvious scratch marks and transverse cracks at *Lc*2. As shown in Table 1, a Ti_0.38_Al_0.62_N-coated tool with a higher *Lc*1 (79 ± 2 N) has a higher cracking resistance. The better adhesion force and toughness of the Ti_0.48_Al_0.52_N coating (105 ± 2 N, 2574 N^2^) reduced the coating delamination during the machining and provided better protection of the substrate.

The coating thickness of Ti_1-x_Al_x_N-coated tools is 3 μm. The substrate material of Ti_1-x_Al_x_N-coated tools is cemented carbide grade K.

### 2.3. Workpiece Testing

**Figure 4:**
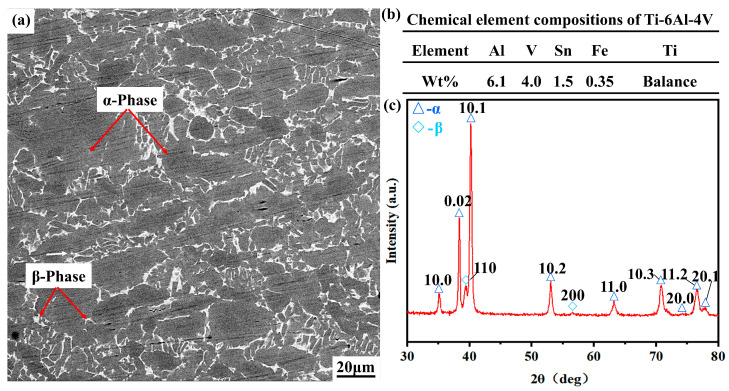
Ti-6Al-4V alloy: (**a**) microstructure; (**b**) chemical element composition; (**c**) XRD spectrum.

The microstructure, chemical composition, and XRD spectrum of the Ti-6Al-4V alloy are shown in Figure 4. Ti-6Al-4V alloy is composed of α-phase and β-phase together, and the α-phase is dominant. Ti, Al, and V are the dominant elements of the Ti-6Al-4V alloy.

### 2.4. Milling Experiment

**Figure 5:**
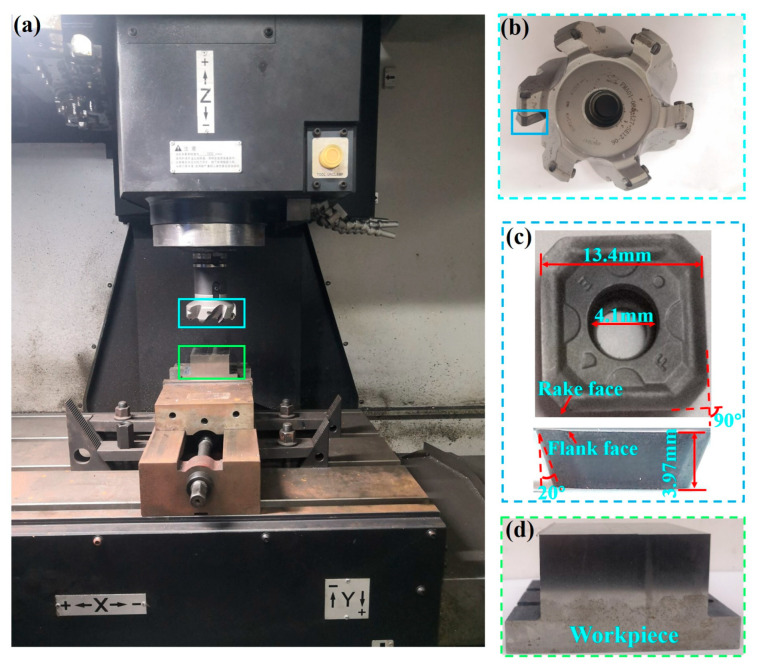
(**a**) Experimental setup; (**b**) milling cutter disc; (**c**) Ti_1-x_Al_x_N-Coated tool; (**d**) Workpiece.

The milling experiments were performed using a Korean vertical CNC machining center DNM-415 (Doosan Machine Tool (China) Co., Ltd., Yantai, China), as shown in Figure 5. The milling cutter disc was selected from FMA01-080-A27-SE12-06 (Zhuzhou Cemented Carbide Cutting Tools Co., Ltd., Zhuzhou, China). The geometry of the Ti_1-x_Al_x_N-coated tool is shown in Figure 5c. γ_0_ and α were 10° after insert installation. The Ti-6Al-4V alloy was machined using dry-down milling. The high-speed cutting parameters for Ti-6Al-4V alloy are given in Table 2. The flank face wear VB = 0.3 mm or notch wear VB_N_ = 0.4 mm was used as the tool failure criterion. The rake face and flank face wear were observed by ultra-deep field 3D observation microscope system (Keyence VHX-5000, Kearns (China) Co., Ltd., Shanghai, China) at different wear stages. SEM and EDS analyses were performed on the tool after the failure criterion was reached to analyze the wear form and the wear mechanism.

## 3. Results and Discussion

### 3.1. Effect of Al Content on Tool Life and Wear Evolution of Ti_1-x_Al_x_N-Coated Tools

**Figure 6:**
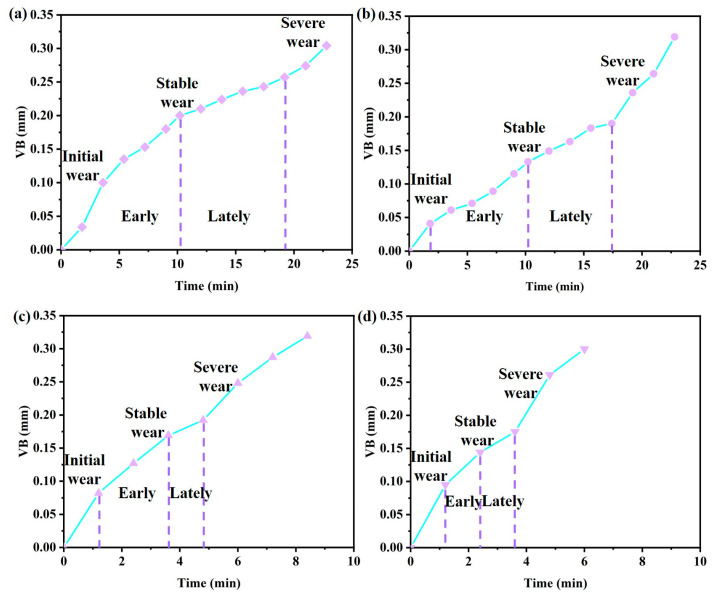
Ti_1-x_Al_x_N-coated tools life curve: (**a**) Ti_0.48_Al_0.52_N-coated tool (*V_c_* = 100 m/min); (**b**) Ti_0.38_Al_0.62_N-coated tool (*V_c_* = 100 m/min); (**c**) Ti_0.48_Al_0.52_N-coated tool (*V_c_* = 150 m/min); (**d**) Ti_0.38_Al_0.62_N-coated tool (*V_c_* = 150 m/min).

**Figure 7:**
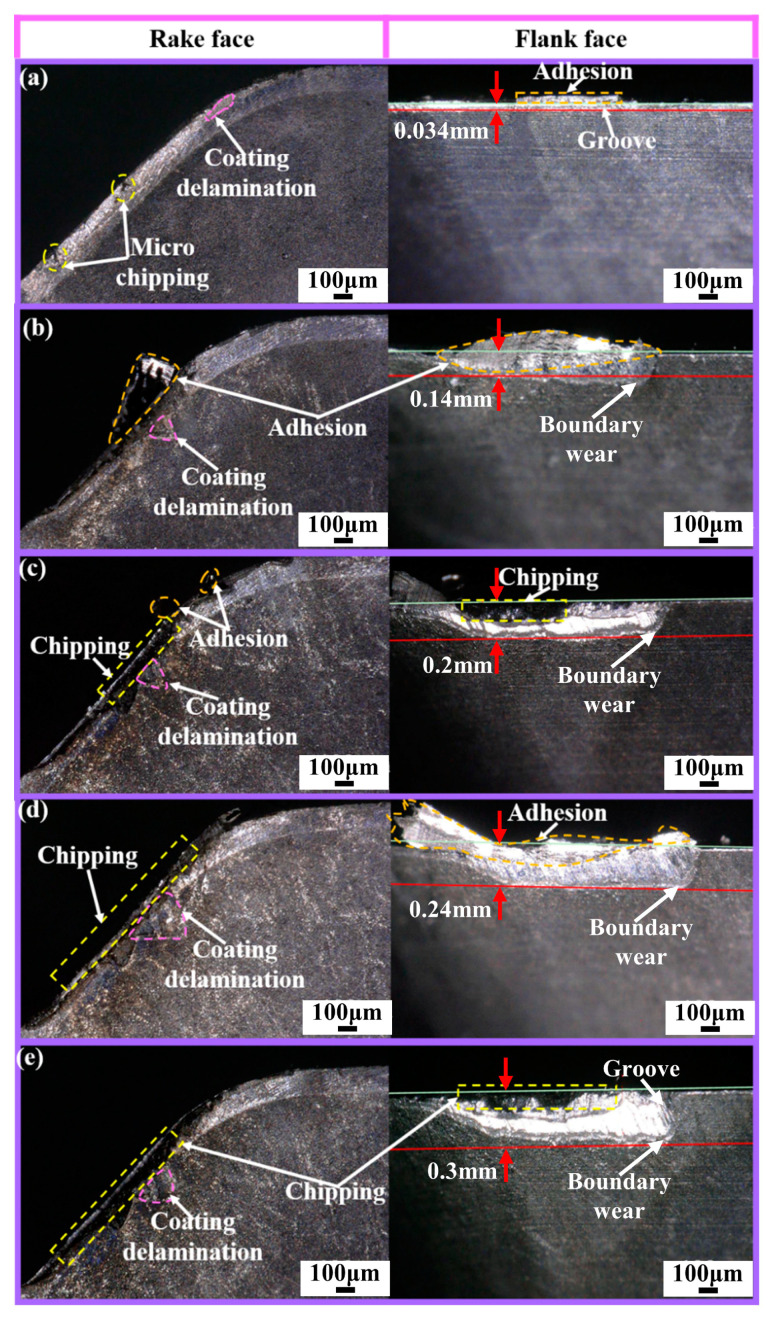
Ti_0.48_Al_0.52_N-coated tool wear evolution (*V_c_* = 100 m/min): (**a**) initial stage; (**b**) early stage stable wear; (**c**) late stage of stable wear; (**d**) early stage of severe wear; (**e**) late stage of severe wear.

**Figure 8:**
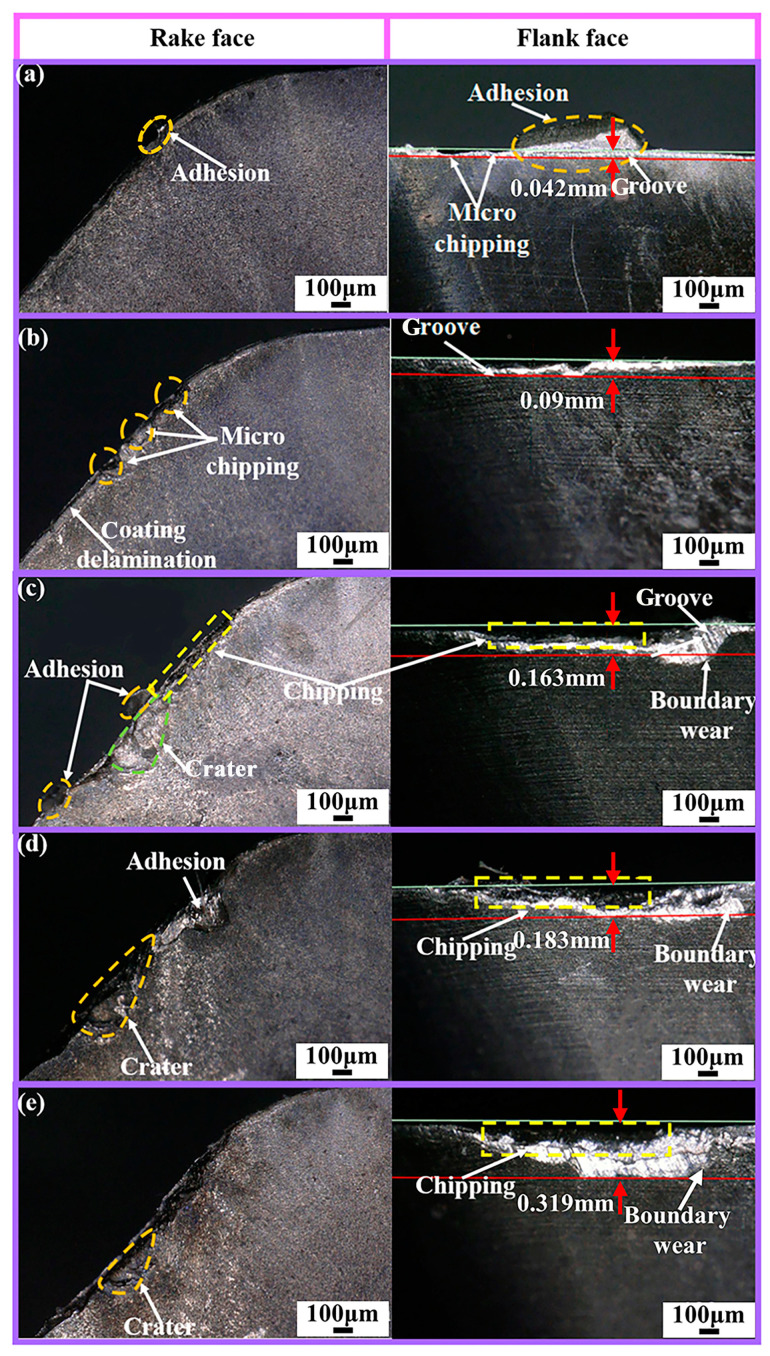
Ti_0.38_Al_0.62_N-coated tool wear evolution (*V_c_* = 100 m/min): (**a**) initial stage; (**b**) Early early stage of stable wear stage; (**c**) Lately late stage of stable wear stage; (**d**) Early early stage of severe wear stage; (**e**) Lately late stage of severe wear stage.

The service life of the Ti_1-x_Al_x_N-coated tools and the wear forms at different wear stages are shown in Figure 6, Figure 7 and Figure 8.

As shown in Figure 6, the Ti_0.48_Al_0.52_N- and Ti_0.38_Al_0.62_N-coated tools have a tool service life of 23 min at 100 m/min. The severe cutting force and cutting temperature at 150 m/min cutting speed led to more serious wear, which made the tool service life relatively shorter. Additionally, the Ti_0.48_Al_0.52_N-coated tool had a longer service life at 150 m/min. Tool wear goes through three stages: the initial wear stage, the stable wear stage, and the severe wear stage.

As shown in Figure 7a, in the initial wear stage, the main wear forms of the Ti_0.48_Al_0.52_N-coated tool were micro-chipping, coating delamination, grooves. At the cutting edge, a large amount of workpiece material adhesion was found. As shown in Figure 7b, the coating delamination was aggravated by the combined effect of the adhered workpiece material and flowing chips. Micro-chipping and adhesion reduced the sharpness of the tool and led to increased cutting force and cutting temperature. At high temperatures, oxidation wear leads to boundary wear. As shown in Figure 7c, severe adhesion, resulting in increased cutting force, made the cutting edge come under increased load, which in turn led to chipping [10]. As shown in Figure 7d,e, the cutting performance deteriorates sharply after chipping occurs in the tool and can rapidly reach the failure criterion.

As shown in Figure 8a, micro-chipping, grooves, and adhesion are the main wear forms in the initial wear stage of the Ti_0.38_Al_0.62_N-coated tool. As shown in Figure 8b,c, a large amount of workpiece material adhering to the tool surface during the stable wear stage leads to an increase in micro-chipping and delamination. In high-temperature and -pressure environments, elemental diffusion occurs between the adhering material and the tool substrate. It resulted in the formation of a crater on the rake face. The presence of the crater weakens the strength of the cutting edge, which in turn leads to chipping. Later in the stable wear stage, boundary wear occurs on the flank face. As shown in Figure 8d,e, the crater wear and boundary wear gradually increase as the cutting progresses, which in turn leads to more serious chipping and causes the tool to reach its failure criterion.

The wear evolution of the Ti_1-x_Al_x_N-coated tools at 150 m/min is not significantly different from that at 100 m/min.

From the above analysis of the wear evolution of Ti_1-x_Al_x_N-coated tools, the rake face changes from initial adhesion and micro-chipping to coating delamination and chipping. The flank face changes from initial adhesion and grooves to boundary wear, build-up layer, and ablation. At the early cutting stage, the main wear mechanisms of tool are abrasive wear, adhesion wear, and oxidation wear. In the later stage of cutting, the main wear mechanisms of tool are adhesion wear, diffusion wear, and oxidation wear.

### 3.2. Analysis of Tool Wear Form and Mechanism When Ti_1-x_Al_x_N-Coated Tools Failure

**Figure 9:**
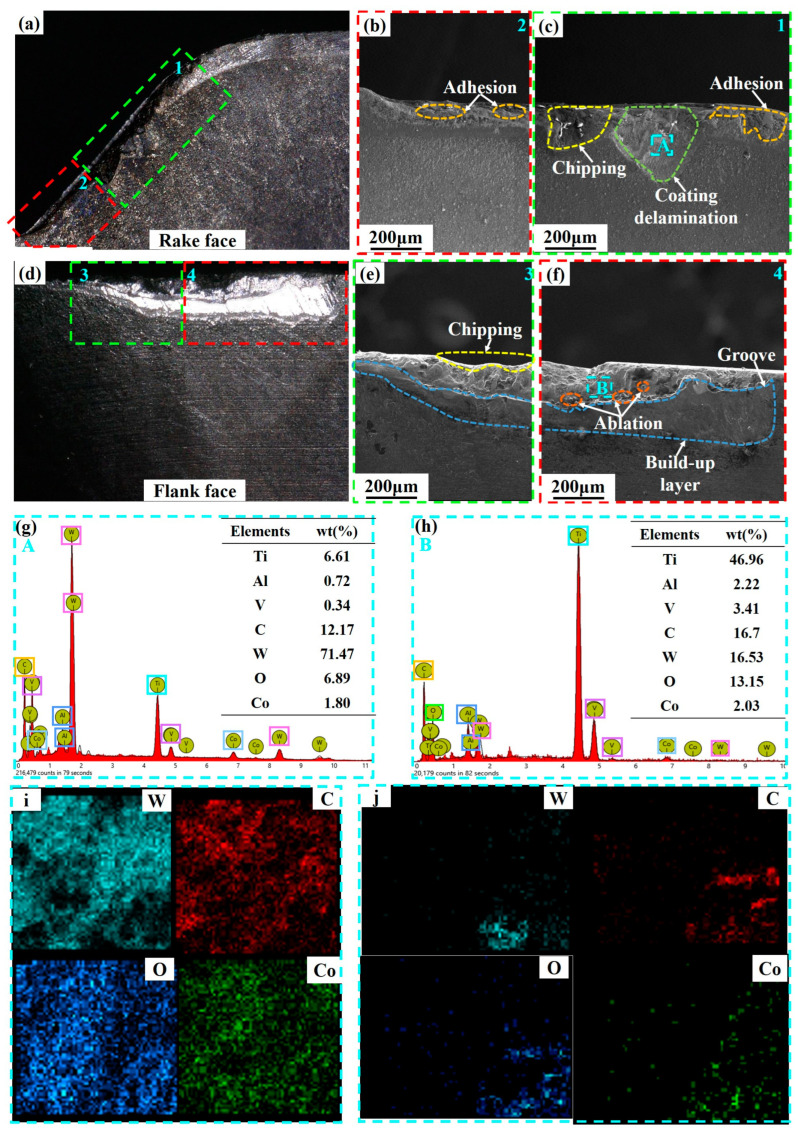
SEM and EDS analysis of Ti_0.48_Al_0.52_N-coated tool wear (*V_c_* = 100 m/min): (**a**) rake face; (**b**,**c**) SEM image of the rake face 1, 2 area; (**d**) flank face; (**e**,**f**) SEM image of the flank face 3, 4 area; (**g**) EDS analysis of the rake face A area; (**h**) EDS analysis of the flank face B area; (**i**) A area element distribution; (**j**) B area element distribution.

Figure 9 shows the SEM and EDS analysis of the Ti_0.48_Al_0.52_N-coated tool when it reaches the failure criterion (VB = 0.3 mm). From Figure 9b,c, it can be seen that adhesion, coating delamination, and chipping are the main wear forms on the rake face. From Figure 9g, the presence of tool substrate elements such as C, W, and Co and workpiece elements of Ti, Al, and V in the A area of the rake face can be seen. This indicates that coating delamination and element diffusion occurred between the tool and the workpiece. In addition to this, the presence of the O elements indicates the occurrence of oxidation wear. As shown in Figure 9e,f, the main wear forms on the flank face were chipping, ablation, build-up layer, and grooves. The analysis in Figure 9h shows that diffusion and oxidation wear also occurred on the flank face. As shown in Figure 9i,j, the distribution of W, C, and Co on the rake face and flank face overlap with O elements and the overlap between Co and O elements were the most serious. This indicates that the oxides are mainly Co oxides. This can reduce the hardness of the tool and make the tool easier to wear.

**Figure 10:**
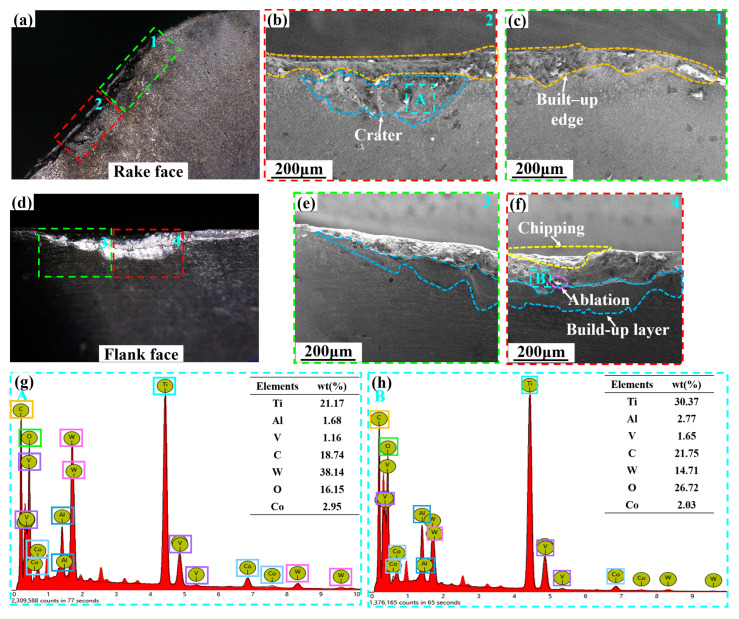
SEM and EDS analysis of Ti_0.38_Al_0.62_N-coated tool wear (*V_c_* = 100 m/min): (**a**) rake face; (**b**,**c**) SEM image of the rake face 1,2 area; (**d**) flank face; (**e**,**f**) SEM image of the flank face 3,4 area; (**g**) EDS analysis of the rake face A area; (**h**) EDS analysis of the flank face B area.

Figure 10 shows the SEM and EDS analysis of the Ti_0.38_Al_0.62_N-coated tool when it reaches the failure criterion (VB = 0.3 mm). As shown in Figure 10b,c, chipping, BUE, coating delamination, and crater were the main wear forms on the rake face. As shown in Figure 10g, elements such as W, Co, Ti, and Al were found in the A area, indicating that element diffusion occurs at the crater. Additionally, diffusion wear was the main cause of the crater formation. As shown in Figure 10e,f, ablation, chipping, and build-up layer were the main wear forms on the flank face. As shown in Figure 10h, the presence of Ti, Co, W, and O elements in the B region indicates the occurrence of diffusion and oxidation wear on the flank face.

As shown in Figure 9 and Figure 10, the Ti and O element content of the flank face were generally higher than that of the rake face, while the W element content was lower than that of the rake face due to serious loss. This indicates more extensive diffusion and oxidation wear on the flank face. The rake face has less adhesion due to chip flow, and diffusion wear is weaker. At the same time, the close contact of the chip makes it difficult for the rake face to contact the air, and the oxidation wear is weaker.

From Figure 9g,h and Figure 10g,h it can be seen that the O elements on the rake face and the flank face of the Ti_0.48_Al_0.52_N-coated tool were weaker than those of the Ti_0.38_Al_0.62_N-coated tool. This indicates that the oxidation wear of the Ti_0.48_Al_0.52_N-coated tool is weaker [34]. In the wear area of the rake face and flank face, the Ti_0.48_Al_0.52_N-coated tool has weaker Ti and Al elements than the Ti_0.38_Al_0.62_N-coated tool, while the W elements is higher than the Ti_0.38_Al_0.62_N-coated tool. This indicates that the diffusion wear is weak for Ti_0.48_Al_0.52_N-coated tools.

As shown in Figure 9c and Figure 10b, it can be seen that the better adhesion force and coating hardness of Ti_0.48_Al_0.52_N coating reduce the damage and coating delamination, and the high H/E and H^3^/E*^2^ enhance the wear resistance of the coating. Therefore, the Ti_0.48_Al_0.52_N coating plays a good role in protecting the tool during the cutting process, which weakens the oxidation and diffusion wear of the tool. As a result, the Ti_0.48_Al_0.52_N-coated tool has a higher service life than the Ti_0.38_Al_0.62_N-coated tool. However, the Ti_0.48_Al_0.52_N coat tool is more suitable for titanium alloy face milling.

**Figure 11:**
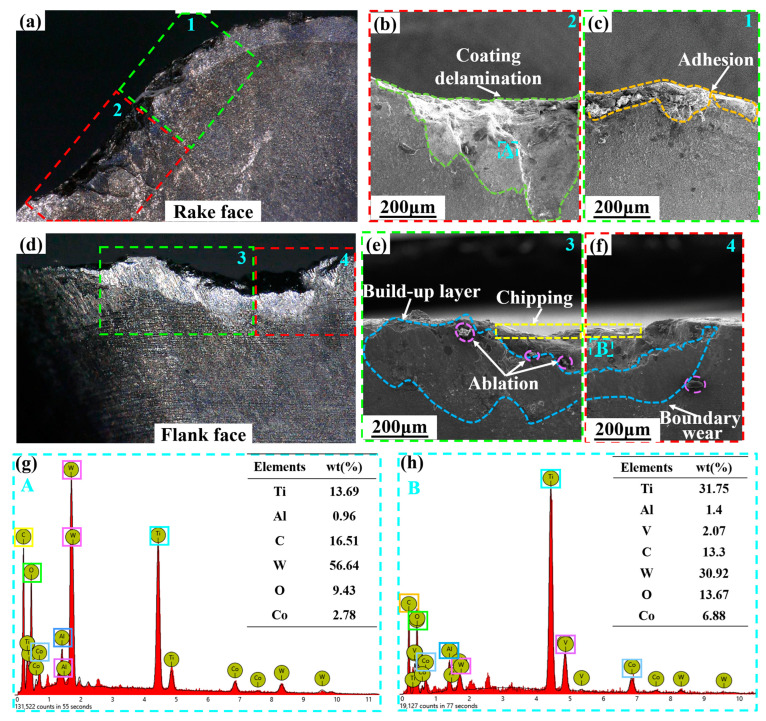
SEM and EDS analysis of the Ti_0.48_Al_0.52_N-coated tool wear (*V_c_* = 150 m/min): (**a**) rake face; (**b**,**c**) SEM image of the rake face 1,2 area; (**d**) flank face; (**e**,**f**) SEM image of the flank face 3,4 area; (**g**) EDS analysis of the rake face A area; (**h**) EDS analysis of the flank face B area.

As shown in Figure 11, adhesion, chipping, coating delamination, ablation, and build-up layer occur in the Ti_0.48_Al_0.52_N-coated tool. The EDS analysis of the A area of the rake face and the B area of the flank face is shown in Figure 11g,h. It can be seen that oxidation and diffusion wear occurred on the tool. Therefore, adhesion, diffusion, and oxidation wear are the main wear mechanisms of the Ti_0.48_Al_0.52_N-coated tool at 150 m/min.

At 150 m/min, Ti_1-x_Al_x_N-coated tools are subjected to greater thermal and mechanical loads, which can cause serious chipping and coating delamination. Additionally, chipping reduces the sharpness of the tool. After the coating delamination, the tool substrate can come into direct contact with the workpiece material and oxygen. This leads to the oxidation of the tool materials and element diffusion, which reduces the strength of the tool. As can be seen from the comparison of Figure 9 and Figure 11, the tool has higher Ti and O elements and lower W elements on the rake face at 150 m/min cutting speed. The cutting temperature increases with the increase in cutting speed. Oxidation and diffusion wear of the tool are aggravated by high temperature. It can be seen that serious oxidation and diffusion wear are the main causes of the tool failure at 150 m/min cutting speed.

As discussed above, it is known that the main wear mechanisms of Ti_1-x_Al_x_N-coated tools are adhesion, diffusion, and oxidation wear. Among them, diffusion and oxidation wear severely limit the tool service life, so it is necessary to further analyze the two wear mechanisms.

**Figure 12:**
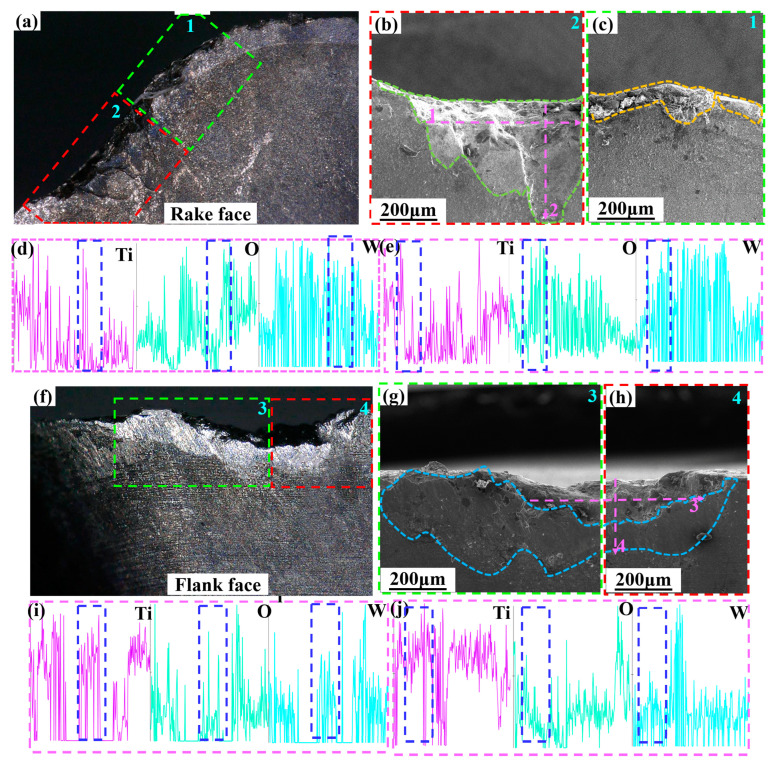
SEM and EDS analysis of Ti_0.48_Al_0.52_N-coated tool wear (*V_c_* = 150 m/min): (**a**) rake face; (**b**,**c**) SEM image of the rake face 1,2 area; (**d**) EDS analysis along the Line 1 scan; (**e**) EDS analysis along the Line 2 scan; (**f**) flank face; (**g**,**h**) SEM image of the flank face 3,4 area; (**i**) EDS analysis along the Line 3 scan; (**j**) EDS analysis along the Line 4 scan.

The EDS line scanning analysis of the wear area of the rake face and the flank face of Ti_1-x_Al_x_N-coated tools is shown in Figure 12. As shown in Figure 12d, along the cutting edge direction, Ti and O elements have higher peaks near the tool tip, while W elements have lower peaks. This indicates that the diffusion and oxidation wear are more serious near the tool tip. As shown in Figure 12e, the peak of Ti and O elements are higher near the cutting edge when it is perpendicular to the cutting edge direction. Therefore, the diffusion and oxidation wear of the rake face are more serious in the area near the cutting edge (near the tool tip).

Figure 12i,j shows the EDS line scanning analysis of the wear area of the flank face. From Figure 12i, it can be seen that along the cutting edge direction, the Ti and O element peaks are higher and the W element peaks are lower near the tool tip. As shown in Figure 12j, along the direction perpendicular to the cutting edge, the Ti element is higher near the cutting edge and the O element has a higher peak near the cutting edge and build-up layer. In summary, it can be seen that the diffusion wear on the flank face is more serious in the area near the cutting edge (near the tip). Additionally, the oxidation wear is more serious in the area near the cutting edge (near the tip) and build-up layer. In Figure 12d,e,i,j, the boxed areas with higher peak Ti and O elements and lower peak W elements correspond to the areas with more severe wear on the rake face and the flank face. Therefore, it is known that severe diffusion and oxidation wear can aggravate the tool wear.

**Figure 13:**
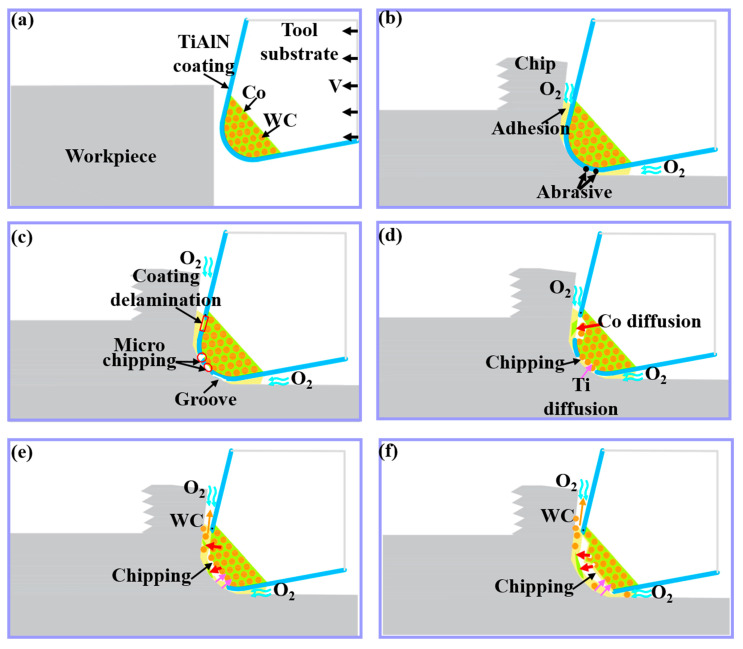
(**a**–**f**) Schematic diagram of Ti_1-x_Al_x_N-coated tool wear evolution process during milling of Ti-6Al-4V alloy.

Based on the analysis of the wear evolution process of Ti_1-x_Al_x_N-coated tools, a schematic diagram was plotted in Figure 13. The wear evolution of Ti_1-x_Al_x_N-coated tools consists of several stages: (i) the chips are adhered to the tool surface under high temperature and high pressure environment, as shown in Figure 13b; (ii) coating delamination, micro-chipping, and grooves, as shown in Figure 13c; (iii) chipping, the element diffusion and oxidation reactions, as shown in Figure 13d,e; (iv) the tool reaches the failure criterion along with a large amount of missing material, as shown in Figure 13f.

## 4. Conclusions

In this paper, the wear evolution of Ti_1-x_Al_x_N-coated tools was explored during the milling of Ti-6Al-4V alloy with Ti_1-x_Al_x_N-coated tools. The effect of Al content and the cutting speed on the wear evolution and wear mechanism of Ti_1-x_Al_x_N-coated tools were analyzed. The conclusions are as follows.

(1) At the speed of 100 m/min, the main wear forms of Ti_1-x_Al_x_N-coated tools on the rake face changed from initial adhesion and micro-chipping to coating delamination and chipping. The main wear forms of Ti_1-x_Al_x_N-coated tools on the flank face changed from initial adhesion and grooves to boundary wear, build-up layer, and ablation. The main mechanisms of tool wear are dominated by adhesion, diffusion, and oxidation wear.
(2) Al content was closely related to the performance of Ti_1-x_Al_x_N coating and seriously affected the form of tool wear and life. The better adhesion force, coating hardness, H/E, and H^3^/E*^2^ enhanced the wear resistance of Ti_0.48_Al_0.52_N coating and reduced its delamination. Therefore, the Ti_0.48_Al_0.52_N coating provides better protection to the tool. This makes the coated tool less wearable and more suitable for titanium alloy milling.
(3) Compared with 100 m/min cutting speed, there was no obvious difference in the evolution of the wear form and wear mechanism of the coated tool at 150 m/min cutting speed. However, the increase in cutting speed aggravated the oxidation wear and diffusion wear, which made the tool fail faster. At the rake face, diffusion and oxidation wear was more serious in the area near the cutting edge (near the tool tip). At the flank face, the diffusion wear was more serious in the area near the cutting edge (near the tool tip). Oxidation wear was more severe in the area near the cutting edge (near the tool tip) and build-up layer.

In this experiment, only two Ti_1-x_Al_x_N-coated tools (x = 0.52, 0.62) were analyzed, and the number of samples was small. In the future, the number of Ti_1-x_Al_x_N-coated tool samples can be increased to further clarify the effect of Al content on Ti_1-x_Al_x_N-coated tool wear. At the same time, it can be considered to add texture to the Ti_1-x_Al_x_N-coated tool surface to explore the influence of texture type, density, and size on tool wear.

## Figures and Tables

**Table 1:** Critical loads and CPRs of Ti1-xAlxN-coated tools.

Ti_1-x_Al_x_N	*Lc*1 (N)	*Lc*2 (N)	*CPRs* (N^2^)
**Ti_0.48_Al_0.52_N**	66 ± 5	105 ± 2	2574
**Ti_0.38_Al_0.62_N**	79 ± 2	102 ± 3	1817

**Table 2:** Cutting parameters.

*V_c_* (m/min)	*ƒ* (mm/z)	*a_e_* (mm)	*a_p_* (mm)
**100, 150**	0.1	10	1

## Data Availability

The data supporting this study’s findings are available from the corresponding author upon reasonable request.

## Abbreviations

BUE	Bulid-up edge
*V_c_*	Cutting speed (m/min)
ƒ	Feed per tooth (mm/z)
*a_e_*	Radial depth of cut (mm)
*a_p_*	Axial depth of cut (mm)
SEM	Scanning electron microscope
XRD	X-ray diffraction
γ_0_	Rake angle
α	Clearance angle
H	Hardness
E	Elastic modulus
H/E	Elastic strain failure capacity
H^3^/E*^2^	Plastic deformation resistance factor

## References

[1] Aufa A.N., Hassan M.Z., Ismail Z. (2022). Recent advances in Ti-6Al-4V additively manufactured by selective laser melting for biomedical implants: Prospect development. J. Alloys Compd..

[2] Xu C., Chen L.Y., Zheng C.B., Zhang H.Y., Zhao C.H., Wang Z.X., Lu S., Zhang J.W., Zhang L.C. (2021). Improved Wear and Corrosion Resistance of Microarc Oxidation Coatings on Ti–6Al–4V Alloy with Ultrasonic Assistance for Potential Biomedical Applications. Adv. Eng. Mater..

[3] Caudill J., Schoop J., Jawahir I.S. (2019). Producing Sustainable Nanostructures in Ti-6Al-4V Alloys for Improved Surface Integrity and Increased Functional Life in Aerospace Applications by Cryogenic Burnishing. Procedia CIRP.

[4] Gupta A., Bennett C.J., Sun W. (2022). High cycle fatigue performance evaluation of a laser powder bed fusion manufactured Ti-6Al-4V bracket for aero-engine applications. Eng. Fail. Anal..

[5] Smart E.F., Trent E.M. (1975). Temperature distribution in tools used for cutting iron, titanium and nickel. Int. J. Prod. Res..

[6] Pramanik A. (2013). Problems and solutions in machining of titanium alloys. Int. J. Adv. Manuf. Technol..

[7] Bobzin K. (2017). High-performance coatings for cutting tools. CIRP J. Manuf. Sci. Technol..

[8] Moreno M., Andersson J.M., Boyd R., Johansson-Jöesaar M.P., Johnson L.J.S., Odén M., Rogström L. (2021). Crater wear mechanism of TiAlN coatings during high-speed metal turning. Wear.

[9] Zhao J., Liu Z., Ren X., Wang B., Cai Y., Song Q., Wan Y. (2022). Coating-thickness-dependent physical properties and cutting temperature for cutting Inconel 718 with TiAlN coated tools. J. Adv. Res..

[10] An Q., Chen J., Tao Z., Ming W., Chen M. (2020). Experimental investigation on tool wear characteristics of PVD and CVD coatings during face milling of Ti 6242S and Ti-555 titanium alloys. Int. J. Refract. Met. Hard Mater..

[11] Wang F., Ji K., Guo Z. (2020). Microstructural analysis of failure progression for coated carbide tools during high-speed milling of Ti-6Al-4V. Wear.

[12] Chowdhury M.S.I., Chowdhury S., Yamamoto K., Beake B.D., Bose B., Elfizy A., Cavelli D., Dosbaeva G., Aramesh M., Fox-Rabinovich G.S. (2017). Wear behaviour of coated carbide tools during machining of Ti6Al4V aerospace alloy associated with strong built up edge formation. Surf. Coat. Technol..

[13] Ziberov M., de Oliveira D., da Silva M.B., Hung W.N.P. (2020). Wear of TiAlN and DLC coated microtools in micromilling of Ti-6Al-4V alloy. J. Manuf. Process..

[14] Yoon S., Kim J., Kim K. (2002). A comparative study on tribological behavior of TiN and TiAlN coatings prepared by arc ion plating technique. Surf. Coat. Technol..

[15] Liu A., Deng J., Cui H., Chen Y., Zhao J. (2012). Friction and wear properties of TiN, TiAlN, AlTiN and CrAlN PVD nitride coatings. Int. J. Refract. Met. Hard Mater..

[16] Wahlström U., Hultman L., Sundgren J.-E., Adibi F., Petrov I., Greene J.E. (1993). Crystal growth and microstructure of polycrystalline Ti1-xAlxN alloy films deposited by ultra-high-vacuum dual-target magnetron sputtering. Thin Solid Film..

[17] Kutschej K., Mayrhofer P.H., Kathrein M., Polcik P., Tessadri R., Mitterer C. (2005). Structure, mechanical and tribological properties of sputtered Ti1–xAlxN coatings with 0.5 ≤ x ≤ 0.75. Surf. Coat. Technol..

[18] PalDey S.C.D.S., Deevi S.C. (2003). Single layer and multilayer wear resistant coatings of (Ti,Al)N: A review. Mater. Sci. Eng. A.

[19] Pemmasani S.P., Valleti K., Gundakaram R.C., Rajulapati K.V., Mantripragada R., Koppoju S., Joshi S.V. (2014). Effect of microstructure and phase constitution on mechanical properties of Ti1−xAlxN coatings. Appl. Surf. Sci..

[20] Ding X.-Z., Samani M.K., Chen G. (2010). Thermal conductivity of PVD TiAlN films using pulsed photothermal reflectance technique. Appl. Phys. A-Mater..

[21] Chen L., Paulitsch J., Du Y., Mayrhofer P.H. (2010). Thermal stability and oxidation resistance of Ti-Al-N coatings. Surf. Coat. Technol..

[22] Kumar K.M., Mathew N.T., Baburaj M. (2022). Sustainable milling of Ti-6Al-4 V super alloy using AlCrN and TiAlN coated tools. Mater. Today Proc..

[23] Uddin M.S., Pham B., Sarhan A., Basak A., Pramanik A. (2017). Comparative study between wear of uncoated and TiAlN-coated carbide tools in milling of Ti6Al4V. Adv. Manuf..

[24] Masooth P.H.S., Jayakumar V., Bharathiraja G. (2020). Experimental investigation on surface roughness in CNC end milling process by uncoated and TiAlN coated carbide end mill under dry conditions. Mater. Today Proc..

[25] Chang K., Dong Y., Zheng G., Jiang X., Yang X., Cheng X., Liu H., Zhao G. (2022). Friction and wear properties of TiAlN coated tools with different levels of surface integrity. Ceram. Int..

[26] Hou M., Mou W., Yan G., Song G., Wu Y., Ji W., Jiang Z., Wang W., Qian C., Cai Z. (2020). Effects of different distribution of residual stresses in the depth direction on cutting performance of TiAlN coated WC-10wt%Co tools in milling Ti-6Al-4V. Surf. Coat. Technol..

[27] Rodríguez-Barrero S., Fernández-Larrinoa J., Azkona I., López de Lacalle L.N., Polvorosa R. (2016). Enhanced Performance of Nanostructured Coatings for Drilling by Droplet Elimination. Mater. Manuf. Process..

[28] Chowdhury M.S.I., Bose B., Yamamoto K., Shuster L.S., Paiva J., Fox-Rabinovich G.S., Veldhuis S.C. (2020). Wear performance investigation of PVD coated and uncoated carbide tools during high-speed machining of TiAl6V4 aerospace alloy. Wear.

[29] Wang F., Zhao J., Li Z., Li A. (2015). Coated carbide tool failure analysis in high-speed intermittent cutting process based on finite element method. Int. J. Adv. Manuf. Technol..

[30] Chang K., Zheng G., Cheng X., Xu R., Li Y., Yu Z., Yang X. (2021). Surface integrity evolution and wear evolution of the micro-blasted coated tool in high-speed turning of Ti6Al4V. Int. J. Adv. Manuf. Technol..

[31] Santana A.E., Karimi A., Derflinger V.H., Schütze A. (2004). The role of hcp-AlN on hardness behavior of Ti1-xAlxN nanocomposite during annealing. Thin Solid Films.

[32] Novák P., Musil J., Čerstvý R., Jäger A. (2010). Coefficient of friction and wear of sputtered a-C thin coatings containing Mo. Surf. Coat. Technol..

[33] Zhang S., Sun D., Fu Y., Du H. (2004). Effect of sputtering target power on microstructure and mechanical properties of nanocomposite nc-TiN/a-SiNx thin films. Thin Solid Films.

[34] Kumar C.S., Urbikain G., de Lacalle L.N.L., Gangopadhyay S., Fernandes F. (2023). Investigating the effect of novel self-lubricant TiSiVN films on topography, diffusion and oxidation phenomenon at the chip-tool interface during dry machining of Ti-6Al-4V alloy. Tribol. Int..

